# Supplementary material for: Evolutionarily conserved ovarian fluid proteins are responsible for extending egg viability in salmonid fish
Source: Sci Rep. 2024 Apr 26;14:9651. doi: 10.1038/s41598-024-60118-2 (PMC11053066; doi:10.1038/s41598-024-60118-2)
Supplement: Supplementary file 1 — Supplementary Legends. [file 41598_2024_60118_MOESM1_ESM.docx]

# Supplementary information

**Table S1**: Proteins identified in Rainbow trout, Atlantic salmon, and Brown trout CF and in Goldfish, Pikeperch and Common carp OF

**Table S2**: Biological processes GO annotations of rainbow trout CF proteome

**Table S3** : Main tissue expression of rainbow trout CF proteins

**Table S4** : Common ancestors and the corresponding proteins specifically enriched in salmonid CF

**Table S5**: Proteins highly abundant or only present in rainbow trout CF fractions with high egg viability preservation potential

**Supplementary figure 1**

A) Replicates of Western blot analysis of N-acetylneuraminic acid synthase a (Nansa) expression in the rainbow trout coelomic fluid. CF samples were collected from 3 separate females and analyzed at 0, 6 and 20 days post ovulation for blots 1 and 2, and at 0 and 20 days post ovulation for blot 3. Blots were cut prior to hybridization. The arrow indicates the sialic acid band at 40kDa. The positions of the molecular weight markers are indicated on the left.

B) Replicates of Western blot analysis of N-acetylneuraminic acid synthase a (Nansa) expression in the rainbow trout ovary. The ovary samples were collected from separate females and analyzed at 0, 6 and 20 days post ovulation for blots 1 and 2, and at 0 and 20 days post ovulation for blot 3. The blots were cut prior to hybridization. The arrow indicates the Nansa band at 40kDa. The positions of the molecular weight markers are indicated on the left.
